# ImmunoDataAnalyzer: a bioinformatics pipeline for processing barcoded and UMI tagged immunological NGS data

**DOI:** 10.1186/s12859-021-04535-4

**Published:** 2022-01-06

**Authors:** Julia Vetter, Susanne Schaller, Andreas Heinzel, Constantin Aschauer, Roman Reindl-Schwaighofer, Kira Jelencsics, Karin Hu, Rainer Oberbauer, Stephan M. Winkler

**Affiliations:** 1 Bioinformatics Research Group, University of Applied Sciences Upper Austria, Softwarepark 13, 4232 Hagenberg im Muehlkreis, Austria; 2 Division of Nephrology and Dialysis, Department of Medicine III, Medical University of Vienna, Waehringer Guertel 18-20, 1090 Vienna, Austria; 3 Department of Biosciences, University of Salzburg, Hellbrunnerstrasse 34, 5020 Salzburg, Austria

**Keywords:** Immunology, Genomics, Next-generation sequencing, Clonality, Diversity

## Abstract

**Background:**

Next-generation sequencing (NGS) is nowadays the most used high-throughput technology for DNA sequencing. Among others NGS enables the in-depth analysis of immune repertoires. Research in the field of T cell receptor (TCR) and immunoglobulin (IG) repertoires aids in understanding immunological diseases. A main objective is the analysis of the V(D)J recombination defining the structure and specificity of the immune repertoire. Accurate processing, evaluation and visualization of immune repertoire NGS data is important for better understanding immune responses and immunological behavior.

**Results:**

ImmunoDataAnalyzer (IMDA) is a pipeline we have developed for automatizing the analysis of immunological NGS data. IMDA unites the functionality from carefully selected immune repertoire analysis software tools and covers the whole spectrum from initial quality control up to the comparison of multiple immune repertoires. It provides methods for automated pre-processing of barcoded and UMI tagged immune repertoire NGS data, facilitates the assembly of clonotypes and calculates key figures for describing the immune repertoire. These include commonly used clonality and diversity measures, as well as indicators for V(D)J gene segment usage and between sample similarity. IMDA reports all relevant information in a compact summary containing visualizations, calculations, and sample details, all of which serve for a more detailed overview. IMDA further generates an output file including key figures for all samples, designed to serve as input for machine learning frameworks to find models for differentiating between specific traits of samples.

**Conclusions:**

IMDA constructs TCR and IG repertoire data from raw NGS reads and facilitates descriptive data analysis and comparison of immune repertoires. The IMDA workflow focus on quality control and ease of use for non-computer scientists. The provided output directly facilitates the interpretation of input data and includes information about clonality, diversity, clonotype overlap as well as similarity, and V(D)J gene segment usage. IMDA further supports the detection of sample swaps and cross-sample contamination that potentially occurred during sample preparation. In summary, IMDA reduces the effort usually required for immune repertoire data analysis by providing an automated workflow for processing raw NGS data into immune repertoires and subsequent analysis. The implementation is open-source and available on https://bioinformatics.fh-hagenberg.at/immunoanalyzer/.

## Background

Lymphocytes play an essential role in the human immune system. Amongst other aspects, lymphocytes protect us from potentially pathogenic microorganisms and cancer cells. An essential aspect of the major lymphocyte types, T and B cells, is the ability of random rearrangements of the variable (V), diversity (D), and joining (J) gene segments of the lymphocyte receptor. [1, 2] This V(D)J recombination is important for the unique antigen receptors such as T cell receptors (TCR) and immunoglobulins (IG). These unique receptors and especially the third complementary-determining region (CDR3) are necessary to recognize and bind different peptides. These peptides are commonly presented by major histocompatibility complexes (MHC) and belong to potentially pathogenic microorganisms or endogenous molecules. [3] V(D)J rearrangement in early T and B cell development contributes to the diversity of the immune system. [4]

Modern sequencing methods allow determining the V(D)J gene segment nucleotide sequences. Next-generation sequencing (NGS) is the current state of the art high-throughput technology for DNA sequencing. The advantages of this methodology, including lower costs and effort, supersede the automated Sanger method [5] in clinical and scientific research. Owing to the increased speed of DNA and RNA sequencing and continuous improvement of read length, usage of such high-throughput systems results in large amounts of data. [6]

Sequencing of the TCR and IG repertoire for deciphering the V(D)J gene segments and CDR3 region allows for the quantitative description of the immune repertoire and its clonal composition. Therefore, several different immune repertoire measures are used in the community as the analysis of clonality and diversity of immune repertoires are of fundamental interest [7]. In addition, these two measures can provide information about the composition of the adaptive immune response. For example, differences in the samples of healthy and diseased individuals can be identified.

### Immune repertoire measures

First, clonotypes are defined as clonally related cells derived from a common progenitor cell. Clonotype count and frequency measures are used for clonality calculations [8]. Within the V(D)J recombination, T cell clones have identical amino acid (AA) sequences of the CDR3 region and identical V and J gene segment pairings. B cells additionally undergo somatic hypermutation (SHM) events. [9, 10] The CDR3 region is a unique or highly similar nucleotide sequence for each T or B cell clone and contributes to the specificity and structure of the TCR or IG. [9] Therefore, the CDR3 regions are of high interest when studying IG and TCR repertoires. Clonality analysis includes quantifying unique CDR3 regions, CDR3 AA length investigation, and examining identical V and J gene segments. Using these measures we can describe immune reactions and offer the potential for monitoring healthy and diseased individuals and innovative treatments. [11] Both CDR3 sequence and CDR3 sequence length may aid in determining the structure and specificity of the TCR or IG. TCR CDR3 sequence specificity, can be analyzed using VDJdb^1^, a TCR sequence database which contains over 42,211 different TCR sequences [12]. Further, responses to an antigen can be described, among other factors, by recognizing changes in the CDR3 lengths and the AA length distribution and over-represented clonotypes. [13, 14]

Second, as an other immune repertoire measure, the diversity describes the heterogeneity of the TCR or IG repertoire. In general, diversity indices are calculated using continuous measures of quantity [15], or more concise, the steadily increasing number of distinct objects in a particular context (here: identical clonotypes). It is estimated that there are about $10^{12}$ different T and B cells in humans [7]. A diverse lymphocyte receptor repertoire is essential in the defense against potentially pathogenic organisms and malignant cells. [16]

Besides clonality and diversity, further crucial measures in immune repertoire analysis are the investigation of the V(D)J gene segments and their pairings. For instance, V and J gene segment pairing analysis can indicate over-represented clonotypes and aberrations in the clonotype fractions. [17] For immune repertoire analysis, each of these measures is of interest for individual samples, but also for comparison of multiple samples.

Multiple-sample analysis and comparison are essential in immunological research and not yet fully automated starting from raw NGS data. A comparison of two or more samples with each other aids in answering scientific questions about quality and characteristic immunological measures. These investigations are, for instance, significant in the case of time-series, longitudinal samples with pre-, within- and post-treatment information, and comparison of individuals or samples. Commonly used methods are pairwise clonotype overlap analysis of samples for the identification of shared clonotypes and quality control in the case of replicates [17, 18]. Unsupervised hierarchical clustering is additionally used for analyzing the similarity among input samples based on aspects of the TCR or IG repertoire, namely clonality, diversity, and V(D)J gene segments. Hierarchical clustering reveals an overview of similarities based on patient or sample characteristics (e.g., treatments).

Furthermore, information about the TCR and IG repertoire analyses are relevant, but the quality of the entire sequencing data should also be investigated. In general, within each sequencing run, sequencing platform-specific adapters with sample indices are attached to the (c)DNA, and these indices are recorded for each read as part of the sequencing process. During de-multiplexing, reads are assigned to their respective sample based on these indices and are commonly written into separate files. Non-assignable reads that cannot be assigned with sufficient accuracy to a specific sample are routinely collected within a dedicated file for undetermined reads. [19, 20] Reasons for unsuccessful assignments can be poor quality of indexing reads (indicated by a low average Phred quality score [21, 22]), missing or erroneous adapter sequences. Investigation of the undetermined reads reveals insight into their composition.

Over the last years, applications of machine learning (ML) have become increasingly important in computational immunology. Applying ML methods promotes the discovery of models that describe the provided dataset and possibly aid in identifying features (e.g., peculiarities in V(D)J pairings) that lead to a specific phenotype [23]. There are several frameworks that are widely accepted and frequently used in this area of research, such as scikit-learn [24], keras [25], HeuristicLab [26], and WEKA [27], e.g. Therefore, the output of data (pre-) processing tools should adhere to the file structure required by these ML frameworks.

In summary, scientists in the immunological field are often forced to invest much time in acquiring basic information about immune repertoire NGS datasets. Therefore, tools which automatically process these datasets and provide the results in a compact summarized format are essential for scientists. When working with TCR or IG data, clonality and diversity are commonly analyzed. Additional interesting components are overlap and similarity analyses to compare multiple samples. While supporting these features, quality control within the whole data processing workflow has to remain traceable.

## Implementation

**Fig. 1:**
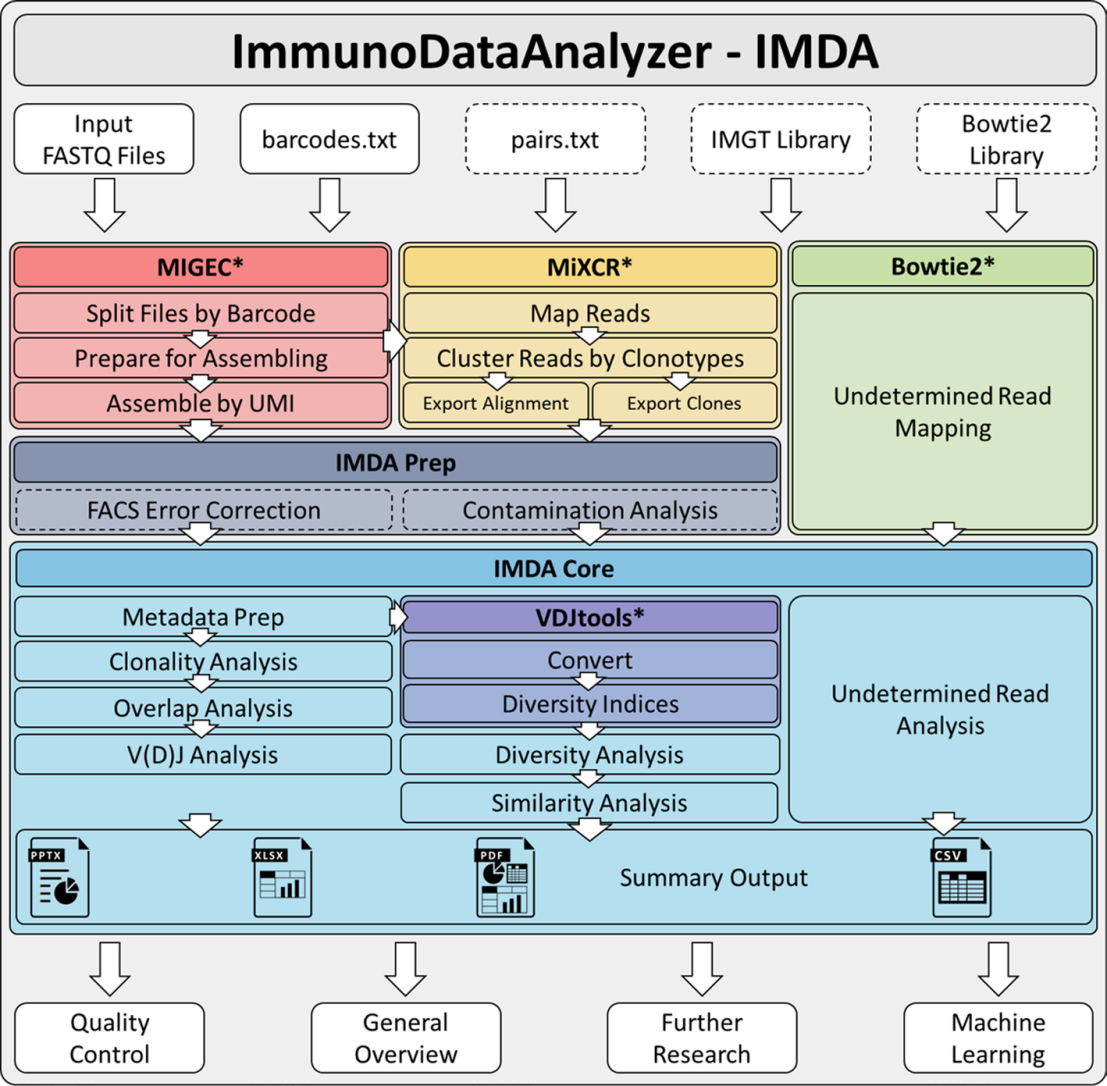
Schematic representation of the IMDA pipeline for automated processing of barcoded and unique molecular identifier (UMI) tagged immune repertoire NGS data starting with input files (compressed or non-compressed FASTQ files) and the barcode file (*barcodes.txt*). Optional files are represented in dashed white boxes. This includes the usage of different library files for better comparability and more efficient performance, e.g., the IMGT library used by MiXCR. Raw data pre-processing using open-source software tools such as MIGEC and MiXCR runs parallel to pre-processing of the undetermined reads. However, *Undetermined Read Mapping* and *Analysis* can only be performed if a Bowtie2 library file is provided. A *FACS Error Correction* module is implemented for cell subset disambiguation within the sub-process named IMDA Prep. If different cell types of one sample separated using FACS or magnetic sorting are sequenced, cell sorting errors can be reduced. The *Contamination Analysis* module enables the identification of shared UMIs within all samples as a measure of quality control in the case of cross-sample contamination. These two IMDA Prep modules are optional (dashed lines) and not mandatory required for IMDA Core analyses. The module IMDA Core provides methods for calculating clonality, diversity, clonotype overlap, sample similarity and V(D)J gene segment analysis, and undetermined read investigations. All data describing the dataset is summarized in a compact format, provides a general overview, enables first interpretations and quality control, and can be used as input for subsequent ML. * MIGEC, MiXCR, Bowtie2 and VDJtools are called and used as third-party tools

ImmunoDataAnalyzer (IMDA) is an automated processing pipeline for immune repertoire NGS data implemented in Python 3.9. It supersedes manual step-by-step processing by providing an automated processing workflow for raw sequencing data. In addition, IMDA produces compact summaries and visualizations describing specific measures and compositions of immune repertoires. Consequently, IMDA provides methods for determining clonality, diversity, and measures for multiple-sample comparison (e.g., clonotype overlap analysis) and allows immediate first interpretations of immune repertoire measures and the sequencing quality. A complete overview of IMDA is shown in Fig. 1.

IMDA comprises four well established open-source software tools for NGS data pre-processing, clonotype assembling, immune repertoire measure calculation, and read mapping to reference sequences:

(1) MIGEC^2^ for read assignment (de-multiplexing) by barcode and unique molecular identifier (UMI) consensus assembling [28], (2) MiXCR^3^ for gene mapping and identification and quantification of clonotypes [29], (3) and VDJtools^4^ for format conversion and calculation of additional diversity indices [30]. (4) Furthermore, Bowtie2^5^ for mapping the undetermined, non-assignable reads on reference genes [31].

IMDA concerts the execution of these open-source tools. Initially, de-multiplexing, UMI consensus assembly and clonotype construction are performed using MIGEC and MiXCR. In IMDA, automated de-multiplexing and UMI clustering are performed by using MIGEC. If this is achieved using other open-source tools, e.g., pRESTO^6^ [32], the IMDA workflow can be started at the MiXCR entry point. MIGEC (and pRESTO) are at present designed for UMI tagged mRNA/cDNA. Thus, usage of MIGEC in IMDA is only recommended for sequenced RNA or cDNA.

After the initial pre-processing phase, results from clonotype construction are used as input for the calculation, evaluation, and visualization of commonly used immune repertoire measures such as clonality, diversity, clonotype overlap, sample similarity, and V(D)J gene segment usage. As part of the pre-processing, automated quality control is performed, and a processing resume is generated. Optional modules for cell subset disambiguation and contamination analysis can be included directly after the pre-processing phase. In Fig. 1, these optional modules are surrounded by dashed lines. Using the cell subset disambiguation module (named *FACS Error Correction*) is intended for removing shared clonotypes from pairs of samples using a frequency fold change criterion. This method is designed for cell fraction cleanup. Thus, it can be used for cells separated according to specific cell characteristics (e.g., cluster of differentiation (CD) antigens—especially CD4^+^ and CD8^+^) using FACS or magnetic sorting to avoid errors in the sorting process. The *Contamination Analysis* based on UMI tagging and identical V(D)J hits reveals information about shared reads within multiple samples. This method is intended to be used if cross-sample contamination is indicated. IMDA further holds functionality for analyzing sequencing reads that could not be assigned to any sample; these functionalities are implemented in the module *Undetermined Read Analysis*. All relevant information is collected and exported in different format, namely presentation, spreadsheet, and tab-delimited files. The presentation file contains essential visualizations generated within the IMDA pipeline for immediate interpretation and data control. The spreadsheet file provides all relevant calculated numeric data. Finally, the tab-delimited file contains sample specific information and can be consumed by commonly used machine learning applications.

**Fig. 2:**
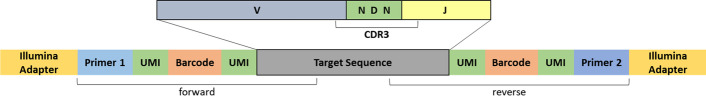
Schematic representation of the sequenced TCR $\beta$ chain region including Illumina adapters, primers, barcodes, UMIs, the V(D)J gene segments, and the CDR3 region. Here, both reads, forward and reverse, include barcodes and UMIs

### Implementation details

IMDA includes five semi-independent processes. The pipeline can be invoked starting from any of these sub-processes once the required input files are available. The execution of each process can be enabled independently of the others according to the users’ requirements. Within IMDA, for each analysis mentioned in Fig. 1, a Python implementation is provided.

**Table 1:** Example for tab-delimited table structure serving IMDA as input adapted from the *barcode.txt* file of the provided test data. The format is analog to the one used by MIGEC. This file should contain for each sample: a sample ID defining the name of the sample and the barcode sequence containing the barcode (here: CAGAT) and optional UMI (represented by “N”). Further, if available, an additional barcode sequence can be defined. Mandatory inputs are the FASTQ files containing all sequencing reads, forward (#1) and reverse (#2)

#Sample ID	Master barcode sequence (barcode and UMI)	Additional barcode sequence	FASTQ #1	FASTQ #2
1_A_nS_r1	NNNNNNtCAGATtNNNNNNtcttgggg		idx1_R1_001.fastq.gz	idx1_R2_001.fastq.gz
1_A_nS_r2	NNNNNNtCAGATtNNNNNNtcttgggg		idx2_R1_001.fastq.gz	idx2_R2_001.fastq.gz
2_A_nS_r1	..		..	..
2_A_nS_r2	.		.	.

The first sub-process performs read de-multiplexing by barcodes that are part of the raw sequencing reads and consensus assembling based on UMIs using the open-source tool MIGEC (see Fig. 1—*MIGEC*, colored in red). For this sub-process, FASTQ files (in compressed or non-compressed format) and an additional text file containing barcode information are required. This text file has to contain the barcode sequences for each sample which can optionally include a UMI region (see Table 1 in “Input formats” section). The second sub-process performs clonotype assembly using the open-source tool MiXCR (see Fig. 1—*MiXCR*, colored in yellow). All commands necessary for constructing clonotypes (nucleotide and AA sequences of CDR3 region and V(D)J gene segments) are automatically executed. Within the third sub-process undetermined reads are mapped to reference genes and genomes using *Bowtie2* (Fig. 1—colored in green). IMDA pre-processing methods implemented in the fourth sub-process named IMDA Prep (Fig. 1—colored in dark blue) include the cell subset disambiguation module named *FACS Error Correction* and the cross-sample contamination analysis method based on UMIs. These methods are optional (surrounded by dashed lines). The last sub-process named IMDA Core (Fig. 1—colored in light blue) performs the actual analysis of the immune repertoires and includes methods for processing, calculating, evaluating, and visualizing the results provided by *MIGEC*, *MiXCR*, and the methods of IMDA Prep as well as the undetermined read mapping results for interpretation.

**Fig. 3:**
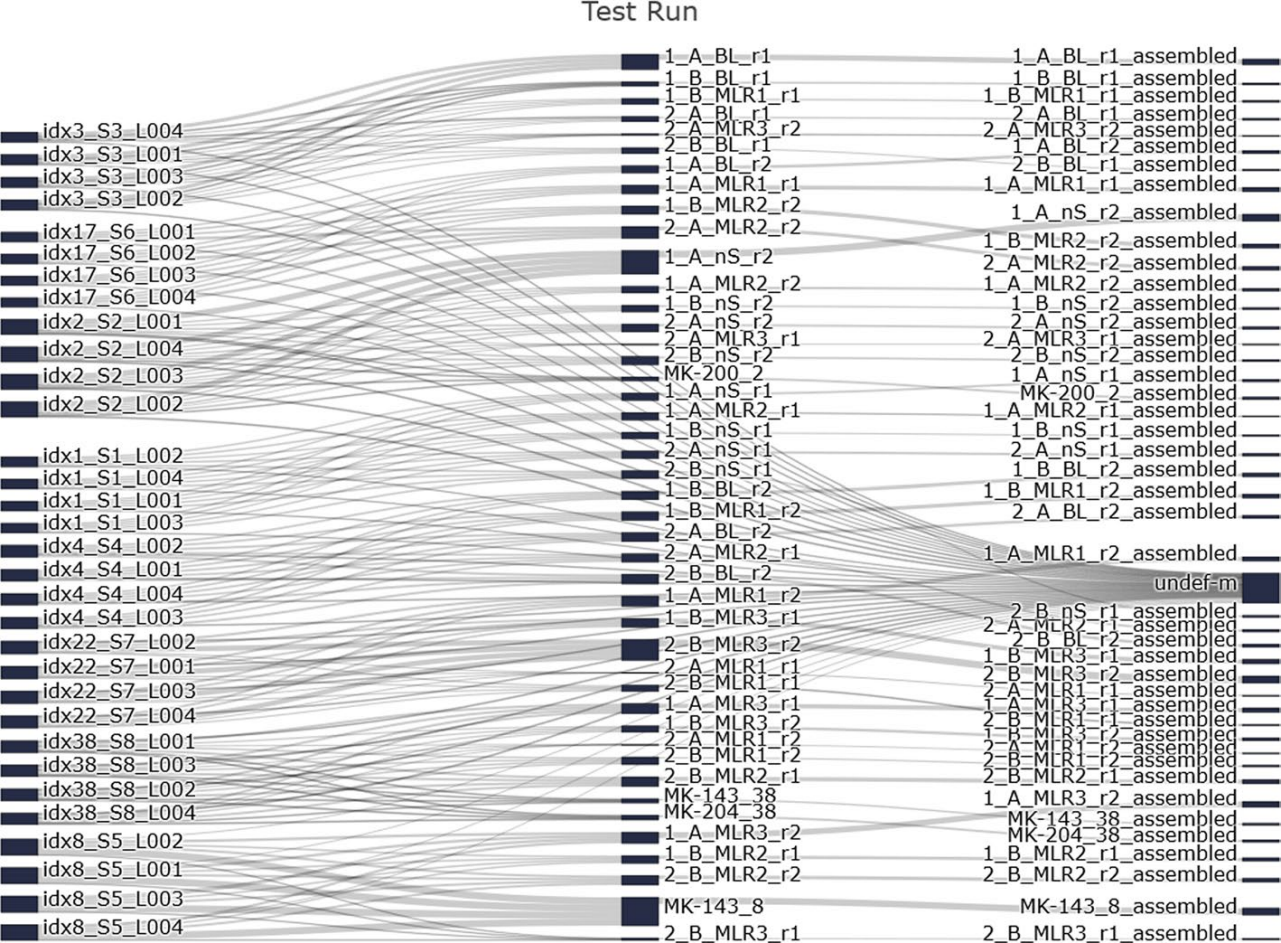
Read assignment visualization of the number of assigned reads after executing MIGEC commands in *MIGEC* sub-process. The left column shows the raw data read counts for each available input file in FASTQ file format. The central column shows the number of reads after de-multiplexing and the right column shows the number of reads after UMI assembling and consensus sequence building. For all files, reads that could not be assigned to a specific sample, due to barcode or primer errors, are assigned to *undef-m* (=undetermined reads)

Clonality, diversity, and clonotype overlap analyses are evaluated based on CDR3 AA sequence counts and frequency calculations. V(D)J gene segment and similarity analyses use the V(D)J gene segment information. IMDA Core further includes the use of the open-source tool VDJtools for calculating multiple diversity indices (Fig. 1—*VDJtools*, colored in violet). In the final step, all results are stored in summary files. These contain all relevant information, including tool settings, read counts, alignment rates, calculations, and visualizations. All relevant results calculated and visualized using IMDA will be described later in “Results and discussion” section.

**Fig. 4:**
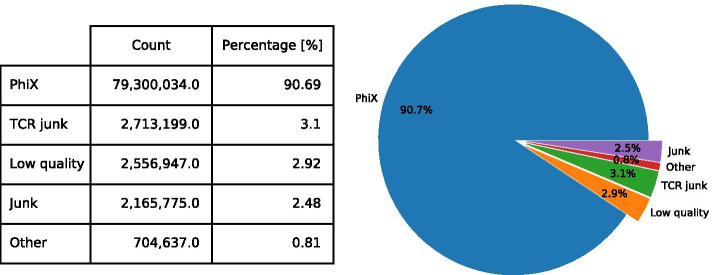
Representation of the IMDA output of the undetermined reads. All reads are assigned to one of the following five groups: *PhiX*, *TCR junk*, *Low quality*, *Junk* reads (which contains reads with consecutive nucleotides in unexpectedly high number) and the group *Other*, whose reads cannot be assigned to one of the other groups. The non-assignable reads within this group *Other* are stored in a FASTQ file providing the possibility for further analysis using, e.g., BLASTn or other sequence alignment tools

IMDA makes use of Python Standard Libraries and, the *SciPy* [33] as well as the *pandas* [34] library for data handling. For visualization we use the *seaborn* [35], *plotly* [36] and *HoloViews* [37] data visualization libraries. For summarizing and providing a compact overview of all calculated and visualized information we use the libraries *python-pptx* [38] and *xlsxwriter* [39] which is integrated in *pandas*. Both, *python-pptx* and *xlsxwriter* provide methods for writing data into a presentation and a spreadsheet file, respectively, which are compatible with Microsoft Office and LibreOffice.

### Input formats

The main input file format for executing the first and second sub-process of IMDA, namely *MIGEC* for de-multiplexing and *MiXCR* for clonotype identification and quantification, are compressed or non-compressed FASTQ files. Additional mandatory input is a tab-delimited file specifying the barcode and UMI sequence for each sample (see Table 1), following the format instructions defined by the open-source tool MIGEC. IMDA reuses this information specified in the barcode file, so no further sample, barcode and UMI description are necessary. By executing the *MIGEC* sub-process using IMDA, suitable files for *MiXCR* in FASTQ file format are generated and automatically processed.

**Fig. 5:**
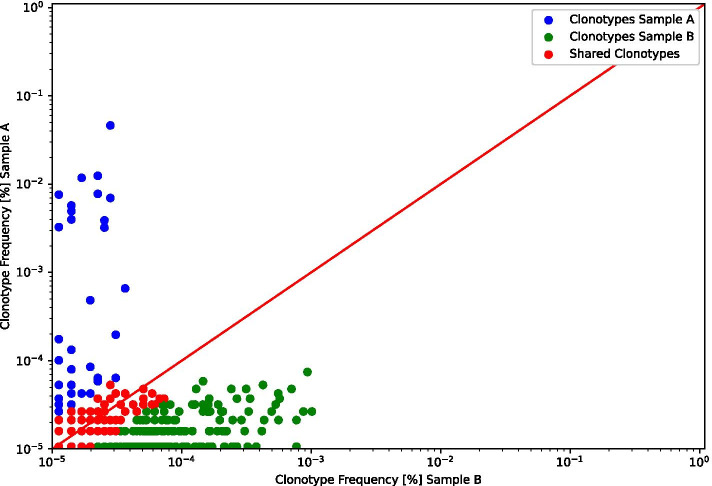
An exemplary representation of shared clonotypes of T cell subsets by clonotype frequency as, for instance, CD4^+^ and CD8^+^ T cells. Blue-colored clonotypes belong to sample A; green-colored clones are T cell clonotypes of sample B. Shared clonotypes (shown in red) are assumed to be in both samples due to inaccurate FACS or magnetic sorting and will be eliminated for further analysis

Optionally, the international ImMunoGeneTics information system^®^ (IMGT^®^) library can be used for alignment and clonotype assembly in *MiXCR* for better comparability with results generated by IMGT/HighV-QUEST [40]. The library is available from https://github.com/repseqio/library-imgt/releases. IMGT/HighV-QUEST is a web based standalone alternative to MiXCR and provides the most complete database for immune repertoire analysis. In IMDA, MiXCR is used because it is a command-line tool, its ease of use, and it offers PCR and sequencing error correction.

If undetermined read analysis is required, the open-source tool Bowtie2 is needed, which requires compressed or non-compressed FASTQ or FASTA files. Furthermore, IMDA allows for the usage of individual Bowtie2 libraries for mapping the undetermined reads. This file can easily be built from a FASTA file, including all sequences on which the undetermined reads should be mapped using the *bowtie2-build* command integrated in Bowtie2.

**Fig. 6:**
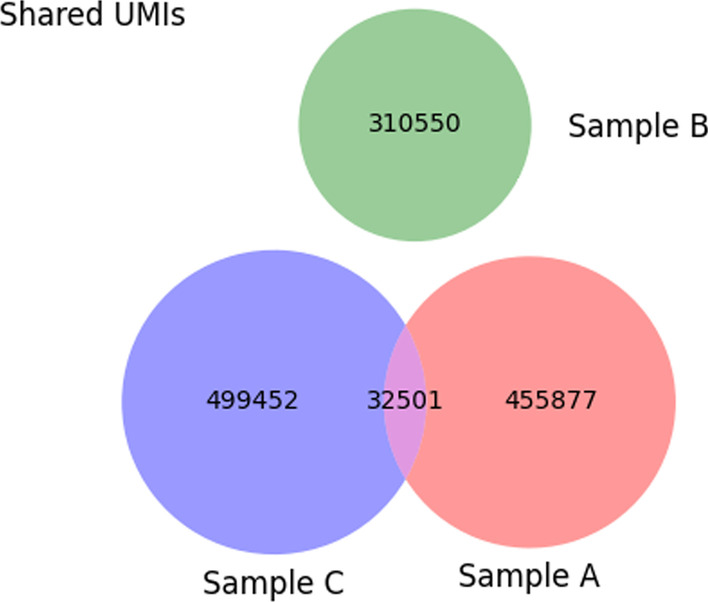
Venn diagram of shared UMIs combined with V(D)J hits for cross-sample contamination detection in two samples. While there are no shared UMI-V(D)J hit-combinations found in samples A and B or sample C and B, samples A and C show an intersection

For using the optional cell subset disambiguation method named *FACS Error Correction*, a tab-delimited text file is required defining pairs of samples that shall be cleaned (see Table 2).

### Usage, pipeline options and method summary

Execution of IMDA is controlled in a single settings file. This file includes all paths and the required methods can be activated or deactivated. As part of the general setup, the paths to the four open-source tools have to be defined. Analysis specific information comprises the paths to the input files (sample and/or undetermined) and the barcode file. If a cell subset disambiguation for e.g., FACS error correction is required, the path to the pairs text file needs to be set. All methods implemented in IMDA are summarized in Table 3.

**Table 2:** Example for tab-delimited table structure defining sample pairs where cell subset disambiguation is required based on the available pairs text file for the provided test dataset

#SampleID1	SampleID2
1_A_nS_CD4^+^	1_A_nS_CD8^+^
1_B_BL_CD4^+^	1_B_BL_CD8^+^

Subsequently, the entire IMDA pipeline can be invoked by running the settings file (*settings.py*).

### Error handling

In IMDA, all entered file directions and file paths are checked for their existence. Furthermore, during its execution, IMDA prints the most important information to the console. Here, possible errors that may occur during the workflow are reported.

## Results and discussion

The usage of the here described ImmunoDataAnalyzer (IMDA) is exemplified using TCR $\beta$ chain sequencing data. In this section, the inputs, visualizations, calculations, and functionality of the IMDA pipeline will be discussed. The following features are covered: the processing of raw data into clonotypes, the analysis of the undetermined reads, FACS error and cross-sample contamination correction, and the calculation of the different metrics for describing and comparing immune repertoires (clonality, diversity, clonotype overlap, V(D)J gene segment usage and repertoire similarity analysis).

**Fig. 7:**
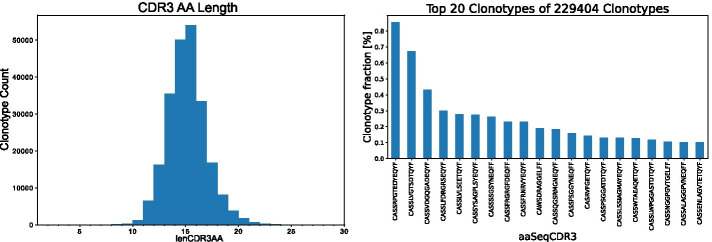
CDR3 AA length distribution (**left**) and top *n* clonotypes (here: $n=20$) of a sample (**right**). Numeric data is provided by MiXCR output and visualized using *ClonalityAnalysis* method implemented in IMDA Core for facilitated interpretation

### Dataset

The here used dataset comprises five apparently healthy volunteers (two female, three male; 23–47 y/o). From those individuals, blood was collected and peripheral blood mononuclear cells (PBMCs) were isolated. One way mixed lymphocyte reactions (MLRs) have been performed to identify an individuals T cells that respond against cells from one of the other potentially human leukocyte antigen system (HLA) mismatched individuals. Two of the five individuals (individuals A and B) were used as responders. The remaining three individuals were used as stimulators. MLRs were carried out separately for each responder-stimulator pair. For each individual, a baseline sample (BL) is available as well as a non-stimulated (nS) sample where no MLR has been performed. In addition to the BL and nS samples, NGS data from MLRs with lymphocytes from three other individuals are provided (MLR1-3) for each individual. NGS TCR $\beta$ libraries were constructed for all T cell bulk samples from all five individuals and the stimulator specific T cells identified in the MLRs. A data subset can be found on our website (https://bioinformatics.fh-hagenberg.at/immunoanalyzer/).

Libraries were sequenced on Illumina NextSeq500. Two sequencing runs with technical replicates were performed (*1_* or *2_* in sample names). The sequencing runs were spiked with Illumina PhiX bacteriophage genome PhiX Control v3 in a concentration of 30% to increase the diversity which is required by these modern NGS machines. [41, 42] Sample de-multiplexing based on Illumina indices was carried out during FASTQ generation and separate FASTQ files were generated for each sample. Resulting reads contain barcodes and UMIs (see Fig. 2). As shown, the region of interest (the TCR $\beta$ chain), is flanked by oligonucleotides including UMI (here: forward and reverse reads contain UMIs), barcode, and primers as well as adapters (here: Illumina adapters), commonly specified by the used sequencing platform. During cDNA synthesis, the nucleotide strands of each individual are tagged with oligonucleotides. [42] These oligonucleotides include the mentioned barcode and UMI. Compared to platform depending indices, the use of additional barcodes introduced directly during cDNA synthesis minimizes the risk of cross-sample contamination as the barcodes are introduced prior to any PCR amplification steps. During the cDNA synthesis and the amplification steps in the PCR, quantitative errors and sequencing errors are possible. [43]

The use of UMIs has multiple advantages. It allows for the quantification of the transcripts, the tracing back of the amplicons to their original RNA, the elimination of PCR errors, and the detection of true variants. [44] Therefore, UMIs are used for making statements about the number of RNA strands whose cDNA was synthesized and amplified successfully during PCR.

It is additionally shown that replicates better correlate when using UMIs and consensus assembling of the reads based on their UMI is performed [45]. UMIs can be further utilized for cross-sample contamination analysis [46]. Shared UMIs in two samples can indicate a cross-sample contamination. If barcodes, as described above, are used, other cross-sample contamination detection methods have to be applied for detecting potential contamination before cDNA synthesis.

If FACS error correction is required, an additional tab-delimited text file defining pairs of samples is necessary. The formats of these two tab-delimited files have been described before in the “Input formats” section.

### Raw data pre-processing

As an initial step, the raw sequencing data in FASTQ file format are processed. IMDA automatically executes MIGEC commands for de-multiplexing and UMI consensus assembling (Fig. 1 - *MIGEC*, colored in red). Subsequently, MiXCR methods are executed to perform clonotype construction for obtaining CDR3 sequences and V, D and J gene segment hits (Fig. 1—*MiXCR*, colored in yellow). MIGEC and MiXCR are executed using *MIGEC* and *MiXCR* sub-processes, respectively. Both tools implement methods for PCR and sequencing error correction. Especially, MiXCR takes special care of clonotypes with identical CDR3 sequence and different V(D)J gene segment sequences to be more robust against sequencing errors [29].

In order to be able to follow the read counts during the read assignment and UMI consensus assembling within *MIGEC* sub-process of the reads better, IMDA generates a Sankey diagram. This Sankey diagram is generated using *plotly* for an (interactive) overview of the read assignments from raw input data up to final assembled reads by UMIs (see Fig. 3) in HTML file format.

**Table 3:** All methods implemented in IMDA (Fig. 1) for automated immune repertoire analysis

Method	Description	Input (I)/Output (O)
MIGEC	Read assignment by barcode (de-multiplexing) and consensus assembling based on UMIs using the open-source tool MIGEC.	I: NGS files in compressed or non-compressed FASTQ file format O: assembled reads in FASTQ file format
MiXCR	Execution of MiXCR commands for clonotype identification and quantification for receiving nucleotide and AA sequences of the CDR3 region and V(D)J gene segments.	I: files in compressed or non-compressed FASTQ file format O: immune repertoire profiling measures (e.g., V(D)J gene segments, CDRs etc.) in text file format
ContaminationAnalysis	Calculates shared UMIs and V(D)J hits of multiple samples for cross-sample contamination analysis.	I: MiXCR output, MIGEC output or non-compressed FASTQ files containing the UMI sequence in the read ID O: cleaned FASTQ files
FACSCorrection	Cell subset disambiguation of clonotypes from cells separated using FACS or magnetic sorting (e.g., CD4^+^ and CD8^+^) and elimination of clonotypes within a twofold change range.	I: filename of pairs for analysis (*pairs.txt*) and MiXCR output O: cleaned files in MiXCR text file format
VDJtools	Executes methods of the open-source tool VDJtools (*convert* and calculate diversity indices) for diversity stats visualization later-on.	I: MiXCR output O: diversity indices for all samples
Bowtie	Analyze undetermined reads from sequencing run and from MIGEC assignment using the open-source tool Bowtie2 for non-assignable read composition analysis.	I: file path to the undetermined files in compressed or non-compressed FASTQ file format and to MIGEC *undef-m* output file, and path to Bowtie2 library O: mapping information is collected in SAM file format
EvaluateUndetermined	If undetermined read analysis using Bowtie2 has been performed, evaluation and visualization of the results is done.	I: the Bowtie2 output in SAM file format O: read counts for each category (see Undetermined Read Processing and Analysis)
ClonalityAnalysis	Clonotype and CDR3 sequence length analysis and visualization is performed.	I: MiXCR output O: CDR3 AA length distribution and clonotype counts
DiversityAnalysis	Diversity curves are calculated and visualized as well as the diversity indices calculated using VDJtools.	I: MiXCR output and VDJtools output O: diversity curves and diversity measures
OverlapAnalysis	Shared clonotype analysis and visualization.	I: MiXCR output O: shared clonotype overlaps (heatmap and LM plots)
SimilarityAnalysis	Hierarchical clustering of all samples is performed and visualized.	I: MiXCR output O: hierarchical clustering information
VDJAnalysis	Calculation of V and J gene segment pairings and visualization using Chord diagrams.	I: MiXCR output O: chord diagrams

### Undetermined read processing and analysis

While raw data is being processed, IMDA allows mapping of the undetermined reads and non-assignable reads from MIGEC checkout assigned to the *undef-m* file on predefined reference genes within a Bowtie2 library (using *Bowtie*—in Fig. 1 colored in green). In this analysis, we used the *bowtie2-build* command to build a reference library containing TCR reference genes and the Illumina PhiX reference genome. The undetermined file(s) contain non-assignable sequences, where the assignment to a specific sample failed because of insufficient accuracy. Bowtie2 provides the mapped reads in SAM file format which then will be evaluated within the IMDA Core module (Fig. 1—light blue).

After all sequences have been processed and mapped with or without success on the reference gene library using the *Bowtie* sub-process and the IMDA Core method *EvaluateUndetermined* is activated, all reads within the SAM output file(s) (undetermined from sequencing and *undef-m* from MIGEC de-multiplexing) are assigned to one of the following five groups: (1) *PhiX*, (2) *Low quality*, (3) *Junk*, (4) *TCR junk*, and (5) *Other*: (1) *PhiX* includes all reads successfully aligned on the PhiX reference genome. (2) If the analyzed read shows a mean Phred quality score lower than 30, the read is assigned to the *Low quality* group. (3) Else, if a read contains consecutive nucleotides in unexpectedly high number (here: number of nucleotide “N” > 10 or more than 1/4 of all nucleotides are “G”), it is assigned to *Junk*. Since the assignment by platform specific index and primer has failed, no biological relevance is assumed but for further investigation these sequences are exported to a FASTQ file. (4) *TCR junk* contains all reads successfully mapped to TCR reference genes which have a mean Phred quality score greater than 30. (5) Reads, which are not assigned to any of the mentioned groups are assigned to the group *Other* and written into a FASTQ file for further investigations using, e.g., BLASTn^7^ [47] or other sequence alignment tools.

In the case of custom reference libraries there is no distinction between the groups *PhiX* and *TCR junk*. All successfully mapped reads are assigned to the same group named *Mapped*.

The analysis of the undetermined or non-assignable reads reveals information about the composition of the undetermined reads. As shown in Fig. 4, the majority of the undetermined reads derived from the sequencing run is assigned to the PhiX reference genome. Another large number of reads is rejected due to its poor Phred quality score. The amount of *TCR junk*, *Junk* and *Other* reads is rather low. The main reasons for an unsuccessful assignment of these reads to a specific sample are absent or erroneous Illumina adapters or barcodes when using MIGEC de-multiplexing. Additionally, the number of reads are compared to the successfully aligned reads by Illumina, where a percentage of about 70 % is expected due to the 30 % PhiX spiked samples.

### Cell subset disambiguation (FACS error correction)

If subsets of T or B cells are separated using FACS or magnetic sorting, inaccurate cell separation can occur. To counteract cell sorting errors, IMDA Prep provides a cell subset disambiguation method (named *FACSCorrection*—colored in dark blue in Fig. 1). Pairs defined within the pairs text file (see format description in the “Input formats” section) are compared and the shared clonotypes are removed. Figure 5 shows the FACS error correction result of two samples. Clones that do not exceed a two-fold change difference between the two samples are considered ambiguously assigned. These clones are visualized (in red) and finally eliminated from both samples for subsequent analysis in IMDA Core. Clones that clearly belong to one of the two samples are shown in green and blue, respectively.

### Contamination analysis

Besides the cell subset disambiguation method named *FACS Error Correction*, IMDA Prep includes a cross-sample contamination analysis based on UMIs and V(D)J gene segment hits (*ContaminationAnalysis*—colored in dark blue in Fig. 1)). Since UMIs are assumed to be unique within simultaneously prepared samples, shared UMIs can be analyzed and indicate cross-sample contamination [46]. Large amounts of sequenced material may exceed the number of possible UMI combinations. Accordingly, in IMDA, UMI sequences of each read are combined with V(D)J hits and are compared between two or more samples. For using this method, the UMI sequence needs to be defined in the header of the reads within the FASTQ files, as shown below. If the IMDA method *MIGEC* is used for consensus assembling of the reads by UMI, FASTQ files in the required format are automatically provided:

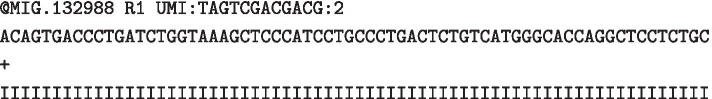



The UMIs are extracted, and the shared UMIs and V(D)J hits are calculated and visualized as Venn diagrams as shown in Fig. 6. Nevertheless, for the dataset used in this study, applying this method is not recommended as this dataset contains data from multiple initial samples that were mixed and sequenced together. To distinguish the samples from each other, the adapters contain UMIs as well as a 5 nucleotides long barcode (Table 1). These 5 nucleotides long barcodes allow to assign reads to a specific sample using MIGEC. On the contrary, the contamination analysis method described in this section is recommended when no additional barcodes are used.

### Clonality analysis

For answering scientific questions it is important to know the clonotypes present in a sample and their frequency. Another important piece of information is the length of the CDR3 AA sequence. Both aspects are relevant for the investigation of the functionality of T and B cells. The CDR3 AA sequence is decisive for the specificity and structure of the TCR and IG, respectively. Information about the clone frequency, V(D)J hits, and CDR3 sequence of a sample are extracted from MiXCR output files. The IMDA Core method *ClonalityAnalysis* generates a histogram of each sample showing the length distribution of the CDR3 AA sequences and visualizes the clone counts of the top *n* clonotypes for better interpretability.

In Fig. 7, an exemplary AA length distribution plot based on the MiXCR output files and the frequency of the top *n* (here: $n=20$) clonotypes are visualized. In this case, the majority of the clonotypes have a CDR3 sequence length of 14 and 15 AAs. The top clonotype accounts for about 0.9% of all 229,404 different clonotypes.

### Diversity analysis

The open-source tool VDJtools provides a comprehensive set of diversity measures for describing the immune repertoire (colored in violet in Fig. 1). These results allow to correlate the immunological status with immune repertoire diversity, compare individuals, and evaluate the number of unique clonotypes. In the step *VDJtools*, VDJtools commands are automatically executed to calculate diversity measures. Furthermore, the diversity analysis approach described in ImmunExplorer (IMEX) [48, 49] can be applied to all samples for calculating and visualizing the diversity curve of each sample by including the *DiversityAnalysis* method.

To improve the comparability of diversity curves between different samples, we standardize their clonotype counts (${n_{scaled}}$ is defined as the lowest number of clonotypes, but is set to 150,000 if the lowest number of clonotypes of one sample falls below 150,000). For receiving the diversity curves an amount of *n* clonotypes (default ${n_{c}}=2500$) of the whole amount of clonotypes is continuously inferred and the unique CDR3 sequences are counted. Parameter optimization is performed using the Python module *optimize* from the *SciPy* library which allows to fit a function *f* to the previously calculated clonotype counts with a stepsize of 2500. The estimated parameters describing the diversity curves and the results of different diversity indices calculated using *VDJtools* can be directly interpreted by the user.

**Fig. 8:**
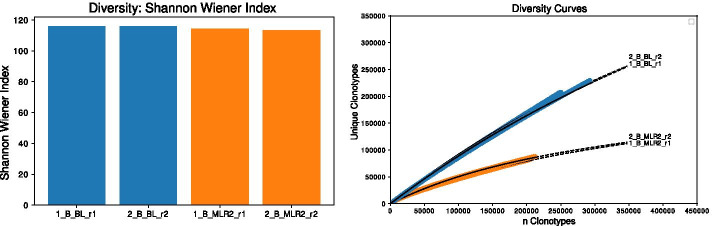
Diversity analysis visualizations of one of the various calculated and estimated diversity indices (left, here: Shannon Wiener index) using VDJtools as well as diversity curves for four samples (right). Technical replicates of the baseline (BL) sample of individual B are shown in blue, technical replicates of the MLR2 sample of individual B are represented in orange

Figure 8 shows an exemplary output of the diversity analysis. The diversity plot (left) allows comparison of the samples using the calculated Shannon Wiener [50] index mean values provided by VDJtools and according to their diversity curves (right). All samples derived from individual B. Technical replicates of the BL samples are shown in blue, technical replicates of MLR2 samples are shown in orange. As expected, the BL samples show a higher diversity than the MLR samples. Diversity analysis using the Shannon Wiener index only shows small differences, but the diversity curves show a clear difference. Since the samples (BL and MLR, respectively) are technical replicates, a high agreement of the curves and diversity indices is desired.

**Fig. 9:**
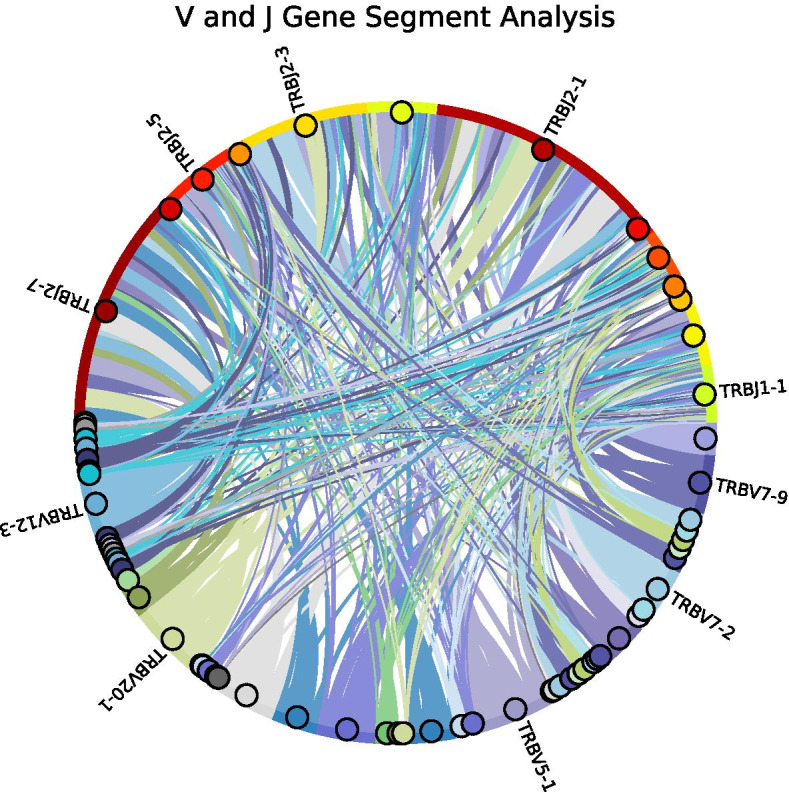
Within the V(D)J recombination, different V and J gene segments are combined. IMDA provides for each sample Chord diagrams for visualizing the clonotype distribution based on V and J gene segment pairs. Every connection represents a V and J gene segment pair. The top *n* (here: *n* = 5) V and J gene segments are labeled

### V–J gene segment analysis

In addition to the diversity analysis and for understanding V and J gene segment usage, Chord diagrams are generated using the visualization library *HoloViews* [37]. These diagrams show the V and J gene segment pair distribution (see Fig. 9). In addition, the widths of the Chord diagram show the proportion of clones containing specific V and J gene segments, respectively. For better interpretability, only V and J gene segment pairs which explain 98 % of all V and J gene segments, are visualized. Additionally, only the top *n* (here: *n* = 5) V and J gene segments are labeled.

V and J gene segment usage is shown in Fig. 9 and shows heterogeneous pairing. For example, this sample’s most occurring V and J gene segment pair is TRBV5-1 and TRBJ2-7. Such Chord diagrams allow for visual identification of over-represented V–J pairings and to compare e.g., expanded V–J pairs in different samples.

**Fig. 10:**
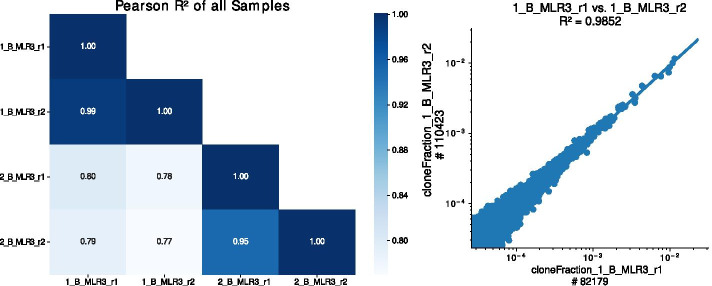
Examples for clonotype overlap output of two samples and their replicates. Left: Correlation matrix of multiple samples compared to each other are plotted as heatmap. Right: LM plot of two samples (replicates) for clonotype overlap analysis

### Clonotype overlap analysis

The clonotype overlap analysis (*OverlapAnalysis*) reveals information about the shared clonotypes between two samples. It allows for detecting errors that occurred during the wet-lab experiments and potential sample contamination. In the case of replicates, clonotype overlap analysis enables to assess library prep reproducibility. IMDA automatically generates linear model (LM) plots visualizing pairwise comparisons of all samples and calculates the correlation given as Pearson $R^2$ values (see Fig. 10). Additionally, a heatmap plot showing the correlations represented as Pearson $R^2$ is plotted using the Python library *seaborn* [35]. The correlation matrix can be found in the spreadsheet summary file generated by IMDA.

Figure 10 shows a subset of the correlation matrix (left) generated as part of the clonotype overlap analysis. The correlation matrix is visualized as a heatmap. The heatmap shows a high accordance within the four biological and technical replicates ($R^2 > 0.75$). An exemplary LM plot of two MLR samples shows the shared clonotypes of these two samples (right). Since the two samples in the LM plot are technical replicates, a high Pearson $R^2$ value was expected.

The clonotype overlap is crucial for detecting contamination and for quality control. Pairwise clonotype overlap analysis can further facilitate identifying expanded clonotypes in response to an immune stimulus (e.g., MLR). By calculating the overlap between BL and MLR samples, expanded clonotypes can be detected. Overlap analysis is especially important for research regarding allosensitization in transplantation as well as vaccination and autoimmune diseases. [17, 51]

### Similarity analysis

In addition to the clonotype overlap analysis, IMDA Core includes the method named *Similarity Analysis* for automated application of hierarchical clustering methods from the *seaborn* and *SciPy* [33] libraries. For reliable interpretation of the results, a standardized approach of the calculated values is needed. Therefore, V and J gene segment frequencies of every sample are used. With the use of hierarchical clustering, we are able to detect contamination, erroneous samples, or sample swaps by grouping the samples by V and J gene segment similarity.

**Fig. 11:**
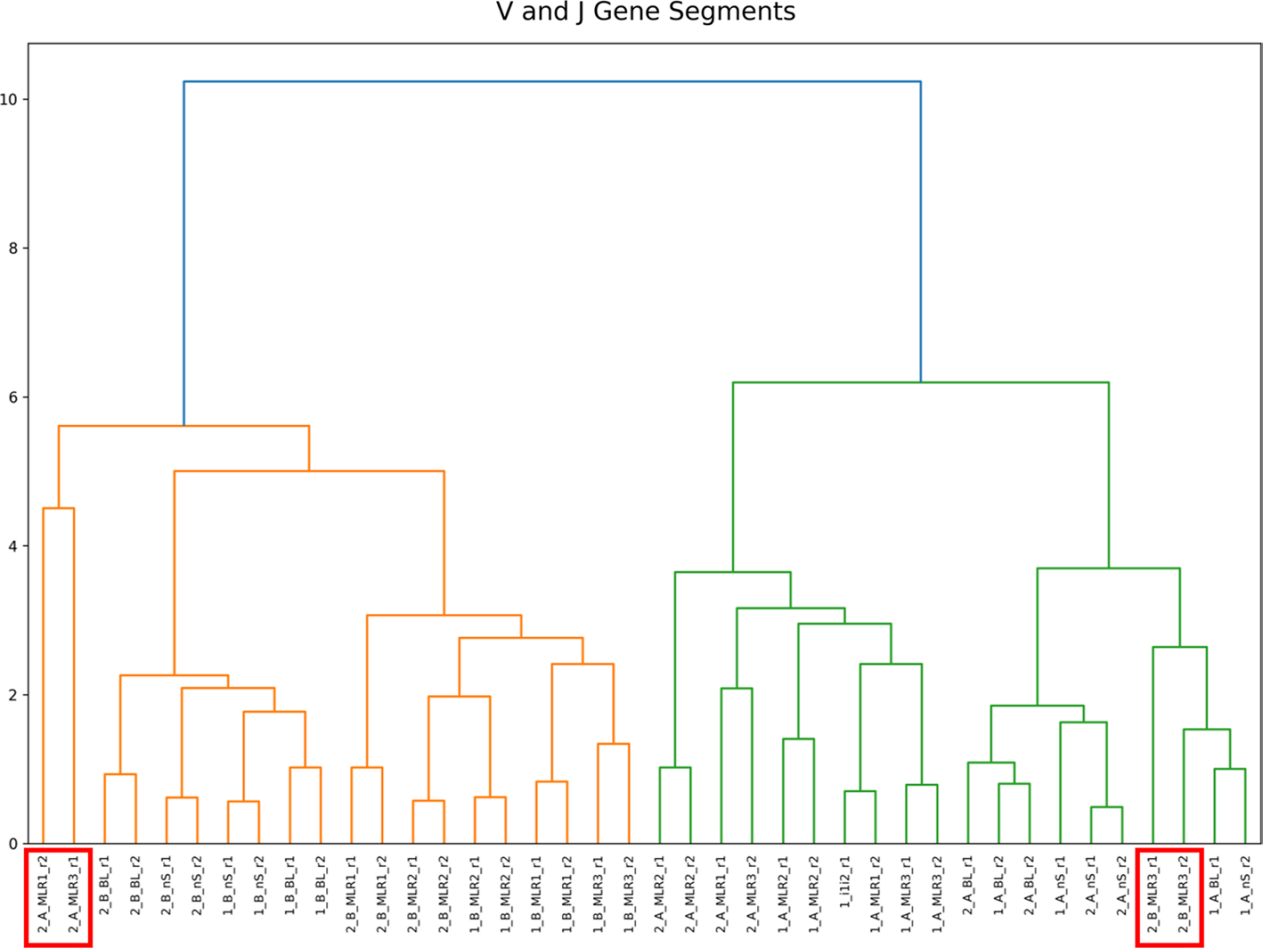
Hierarchical clustering is used for similarity analysis. The graphic shows the similarity of the samples according to V and J gene segment frequencies. Standardization within the columns (samples) is done for accurate comparability. The samples can be divided into two groups—individual A (green) and B (orange). According to this results, four samples (*2_A_MLR1_r2*, *2_A_MLR3_r1*, *2_B_MLR3_r1*, *2_B_MLR3_r2*) should be excluded, since mishaps during laboratory work potentially occurred

Through using the V(D)J gene segment frequencies and the hierarchical clustering approach (see Fig. 11) we show the similarity and grouping of all samples. As demonstrated, through clustering the samples according to their V and J gene segment frequencies, the samples can be divided into two groups—individual A (green) and individual B (orange). However, four samples (*2_A_MLR1_r2*, *2_A_MLR3_r1*, *2_B_MLR3_r1*, *2_B_MLR3_r2*) stand out because they do not belong to the individual to whom they were assigned according to their V(D)J similarity. This is due to a barcode swap. This example confirms that by using hierarchical clustering immediate first interpretations can be performed and mishaps during laboratory workflow can be discovered.

### Summary output

Throughout the processing and analyzing procedure of the data, well-selected information is collected and written to a spreadsheet file. Included are the following information:

- important sample information like barcodes and UMI definitions,
- read counts and trend of the read counts,
- alignment rates,
- clone counts,
- average CDR3 AA sequence length and standard deviation,
- diversity calculations including different diversity indices and curve parameter description,
- a clonotype overlap matrix with the calculated Pearson $R^2$ values for all samples,
- V, D, and J gene segment frequencies,
- and the used commands for the included open-source software tools.

Most relevant plots are collected in presentation file format for an immediate overview, quality check, first interpretations, and further research steps.

### Data export for machine learning

Additionally, the pipeline provides a tab-delimited file (*ml.csv*) which contains selected key features for each sample and can be used as input for ML approaches. This file includes the diversity indices, the diversity curve parameters, and the V(D)J gene segment counts. For more comparable results, the V(D)J frequency values are written to a second tab-delimited file (*ml_norm.csv*). These files can be used for unsupervised ML algorithms (e.g., clustering algorithms) and for supervised learning algorithms (e.g., classification or regression algorithms). An additional column defining the target has to be added for labeling the provided data for supervised learning algorithms. We recommend using the normalized data as input for algorithms implemented in the *scikit-learn* [24] or *keras* [25] as well as for software tools providing a user interface for non-programmers like Weka [27] and HeuristicLab [26].

**Fig. 12:**
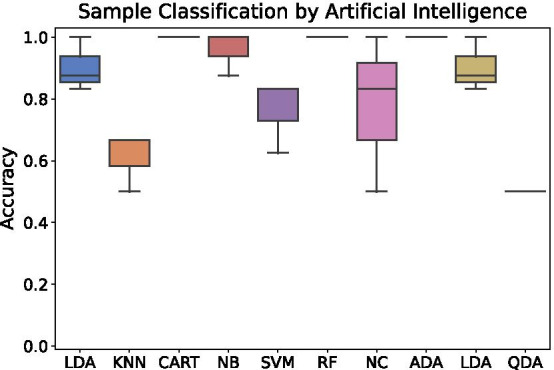
Threefold cross-validation accuracies of the classification of the provided samples according to sample affiliation (individual A or B) using different classification algorithms provided by the Python library *scikit-learn*. *LDA* Linear Discriminant Analysis, *KNN* k-nearest neighbors classifier, *CART* decision tree classifier, *NB* Gaussian Naive Bayes, *SVM* support vector machine, *RF* random forest classifier, *NC* nearest centroid classifier, *ADA* AdaBoost classifier, *LDA* linear discriminant analysis, *QDA* quadratic discriminant analysis

For demonstration, we applied several classification algorithms of the Python library *scikit-learn* on the data discussed before. The target variable is the correspondence to individual A or B. In Fig. 12 we visualized the accuracies of the different classification algorithms. Algorithms such as the decision tree classifier, random forest, and AdaBoost classifier were able to assign all samples correctly and achieve an average accuracy of 100 % in three-fold cross-validation. All three algorithms are based on decision trees, which means if one V or J gene segment occurs only in one of the two individuals, all samples can be classified correctly.

## Conclusions

The calculations and visualizations provided by our ImmunoDataAnalyzer (IMDA) cover a wide range of crucial aspects of TCR and IG repertoires. IMDA allows automated processing and evaluation of immune repertoire NGS data. It supports the processing of barcoded and UMI tagged NGS data. IMDA is built around well-established open-source tools (MIGEC, MiXCR, VDJtools, Bowtie2) and automatizes their execution and thus alleviates NGS immune repertoire data analysis. Furthermore, IMDA comes with cross-sample contamination analysis and cell subset disambiguation methods that are not available elsewhere and automatically provides multiple-sample comparison results.

The IMDA pipeline supports compressed or non-compressed FASTQ files. In the first two steps, *MIGEC* and *MiXCR*, open-source software tools are used for primer trimming, barcode and UMI extraction, consensus assembling (MIGEC), and reconstruction of the actual clonotype sequences (MiXCR). Using MIGEC, IMDA offers the opportunity to process batches of files and IMDA Core methods provide information about relations and differences between the input samples. The tools used are firmly anchored in the immunologic community and are state of the art bioinformatics tools for studying the adaptive immune system. Using IMDA, it is no longer necessary to perform consecutive manual execution of MIGEC and MiXCR commands. Provided results are automatically aggregated and the read counts, alignment rates, and all other information listed in the “Summary output” section are extracted from intermediary results. They are written into a single spreadsheet summary file.

In addition to the automated pre-processing, the undetermined reads are processed and mapped to reference sequences supplied as a Bowtie2 library. Undetermined read analysis allows the detection of potential contamination, aberrations during the sequencing run and describes the composition of the undetermined reads.

For additional data cleaning, IMDA provides within the IMDA Prep module two methods: FACS error correction method for the elimination of shared clonotypes of two samples after FACS or magnetic sorting (e.g., CD4^+^ and CD8^+^ cell separation) and contamination analysis method, providing information about shared UMIs combined with V(D)J hits within all samples.

The core of the IMDA pipeline is the evaluation of the pre-processed data. This includes relevant measures for the immunologic community: clonality, diversity, and clonotype overlap analysis in the case of replicates, time-series, or other comparable aspects. Additionally, visualizations of the similarity of the samples according to their V and J gene segments and their diversity are provided. Furthermore, sample comparison can be made regarding the provided Chord diagram information of the V and J gene segment pairings, allowing first interpretations of over-represented or extended use of specific V and J gene segments. This evaluation and preparation for interpretation are done automatically after the pre-processing. All output files, calculations, and results generated during the process are reported, stored, and available for further custom analyses, validation, and investigations. By providing results of the most crucial aspects of the immunologic field, IMDA supports identifying specific patterns in IG and TCR repertoires.

In summary, IMDA is a bioinformatics framework for quality control and processing immune repertoire NGS data providing the user a broad overview. Samples can be processed from raw data to a well-selected set of key measures and explanatory figures in one go using the contiguous IMDA pipeline. In addition, the evaluation module of IMDA can also be used independently of the *MIGEC* and *MiXCR* sub-processes for analyzing clonotype tables obtained elsewhere.

The IMDA pipeline provides a great overview regarding the CDR3 region, the V(D)J gene segments, and the similarities among samples. Hence, IMDA is perfect for evaluating immunologic NGS data and planning further research steps since all calculations and visualizations are summarized in two compact output files. Furthermore, by investigating the output information, it is further possible to improve the laboratory effort. An additional feature is the third summary file which contains the V and J gene segment information, the diversity indices, and curve parameters and serves as input for various ML methods. In conclusion, IMDA automatically processes FASTQ files and evaluates CDR3 and V(D)J specific measures, summarizes all information, visualizations, and calculations for providing a general overview and provides insights into possible sources of error and gives inspiration for further research. Thus, the most significant advantage of IMDA is providing a good overview of immune repertoire NGS data in an efficient way.

## Availability and requirements

Project name: ImmunoDataAnalyzer (IMDA); Project home page: https://bioinformatics.fh-hagenberg.at/immunoanalyzer/; Operating system(s): Windows and Linux OS (64-bit); Programming language: Python; Other requirements: Python 3.7 or higher, Java 1.8.0, Perl 5.12.3 or higher; License: see License Agreement on IMDA website https://bioinformatics.fh-hagenberg.at/immunoanalyzer/; Any restrictions to use by non-academics: None.

## Data Availability

The most recent version of IMDA, source code, documentation and a test data subset are available online: https://bioinformatics.fh-hagenberg.at/immunoanalyzer/.

## Abbreviations

AA: Amino acid
BL: Baseline
CDR3: Third complementarity-determining region
FACS: Fluorescence-activated cell sorting
HLA: Human leukocyte antigen system
IG: Immunoglobuline
IMDA: ImmunoDataAnalyzer
IMEX: ImmunExplorer
IMGT: International ImMunoGeneTics information system
LM plot: Linear model plot
ML: Machine learning
MLR: Mixed lymphocyte reaction
NGS: Next-generation sequencing
nS: Non-stimulated
PBMC: Peripheral blood mononuclear cells
TCR: T cell receptor
UMI: Unique molecular identifier

## Footnotes

1 available at https://vdjdb.cdr3.net/.

2 available at https://github.com/mikessh/migec.

3 available at https://github.com/milaboratory/mixcr.

4 available at https://github.com/mikessh/vdjtools.

5 available at https://github.com/BenLangmead/bowtie2.

6 available at https://bitbucket.org/kleinstein/presto/downloads/.

7 available at https://blast.ncbi.nlm.nih.gov.
